# Supplementary material for: Virtual-photon-mediated spin-qubit–transmon coupling
Source: Nat Commun. 2019 Nov 6;10:5037. doi: 10.1038/s41467-019-13000-z (PMC6834620; doi:10.1038/s41467-019-13000-z)
Supplement: Supplementary file 1 — Supplementary Information [file 41467_2019_13000_MOESM1_ESM.pdf]

Supplementary Information for the Article: Virtual-photon-mediated spin-qubit–transmon coupling

A. J. Landig et al.

SUPPLEMENTARY NOTE 1: ADDITIONAL MEASUREMENTS

Flux dependent RF spectrum

We characterize the microwave response of coupling resonator, read-out resonator and transmon by measuring the spectrum of the reflected amplitude $|S_{11}|$ in Supplementary Fig. 1. The RX qubit is detuned energetically for this measurement. As Φ_C is produced by a coil mounted next to the sample, it affects both the SQUID array (coupling) resonator and transmon transition frequencies. The transmon is visible in Supplementary Fig. 1 at around its maximum frequency of $\nu_T \simeq 5.4\,\text{GHz}$. It has a faster periodicity in Φ_C than the coupling resonator, as the area of the resonator SQUID loops is smaller compared to the area of the transmon SQUID. We observe about one periodicity of the coupling resonator in Supplementary Fig. 1 with a maximum frequency at $\nu_C \simeq 6.8\,\text{GHz}$. The characteristic impedance of the resonator is frequency dependent as $Z_0 \propto 1/\nu_C$. We estimate $Z_0 \simeq 1.1\text{k}\Omega$ at its maximum frequency. The $50\,\Omega$ (read-out) resonator is resonant at $\nu_R \simeq 5.6\,\text{GHz}$. It is indirectly influenced by Φ_C via the dispersively coupled transmon.

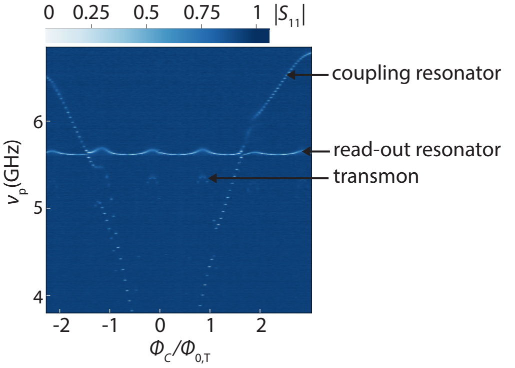

Supplementary Figure 1. Flux dependent microwave response. Spectrum of reflected amplitude $|S_{11}|$ as a function of probe frequency ν_p and flux Φ_C, which is normalized by the transmon flux periodicity $\Phi_{0,T}$. The RX qubit is detuned energetically. Exemplary positions of coupling resonator, read-out resonator and transmon are indicated with arrows.

Extraction of RX qubit parameters

We use two-tone spectroscopy to determine the RX qubit tunnel couplings t_l and t_r. As illustrated in Supplementary Fig. 2a, we apply a resonant probe tone to the coupling resonator ($\nu_p = \nu_C$), a drive tone at ν_{dRX} to a TQD gate electrode and sweep the RX qubit energies with ϵ and Δ. The resulting resonator reflection $|S_{11}|$ is shown in Supplementary Fig. 2c for two different drive frequencies, which are well below ν_C.

The RX qubit asymmetry parameter ϵ quantifies the energy difference of the $(2,0,1)$ and $(1,0,2)$ charge configurations illustrated in Supplementary Fig. 2b. In addition to Δ, that was introduced in the main text, ϵ allows to tune the qubit frequency ν_{RX}. For symmetric tunnel couplings $t_l = t_r$, ν_{RX} has a minimum at $\epsilon = 0$. The minimum position is shifted for asymmetric tunnel couplings [1, 2]. Except for the measurements presented in Supplementary Fig. 2, we operate the RX qubit at this energy minimum in ϵ to protect it from charge noise to first order. Thereby, the minimum position is determined with two-tone spectroscopy [c.f. Fig. 1e].

We observe spectroscopic evidence of the RX qubit in Supplementary Fig. 2c as a reduction in S_{11} whenever

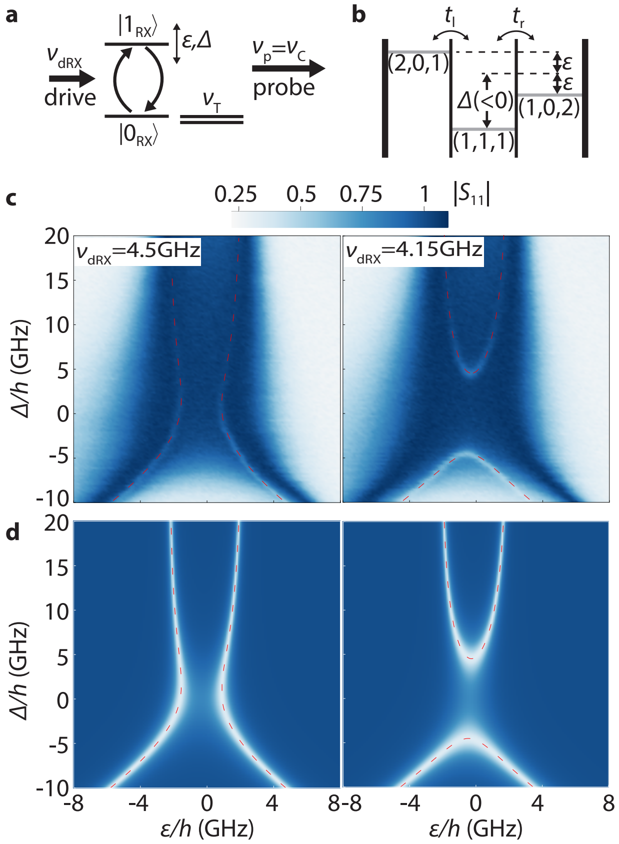

Supplementary Figure 2. RX qubit two-tone spectroscopy. **a** Schematic of the measurement scheme used in **(c)**. RX qubit and transmon are detuned energetically. RX qubit transitions (black arrows) are driven at frequency ν_{dRX}, a probe signal is applied on resonance with the coupling resonator ($\nu_p \simeq \nu_C$). **b** TQD state diagram as in Fig. 1d, showing in addition the left-right dot asymmetry ϵ. **c** Amplitude $|S_{11}|$ of the reflected microwave probe tone at $\nu_p \simeq \nu_C = 4.84\,\text{GHz}$ as a function of RX qubit detuning Δ and asymmetry ϵ for $\nu_{dRX} = 4.5\,\text{GHz}$ and $\nu_{dRX} = 4.15\,\text{GHz}$ in the left and right panels, respectively. The dashed line shows the bare RX qubit energy contour for qubit tunnel coupling configuration 3 and $\nu_{RX} = \nu_{dRX} = 4.5\,\text{GHz}$ in the left and $\nu_{RX} = \nu_{dRX} = 4.15\,\text{GHz}$ in the right panel. **d** Single drive tone simulations of **(c)**. The dashed lines in **(c)** and **(d)** are identical.

drive tone and RX qubit are on resonance ($\nu_{\mathrm{dRX}} = \nu_{\mathrm{RX}}$). We fit $\nu_{\mathrm{RX}}(\epsilon, \Delta)$ calculated from the RX qubit Hamiltonian [1] to the spectroscopic positions to extract t_{l}, t_{r} as well as the lever arms α_ϵ and α_Δ. Thereby the quality of the fit is improved by performing a simultaneous fit to two datasets with different ν_{dRX}. We use a single-tone simulation explained in Supplementary Note 2 to calculate the theoretical response due to a single drive tone in Supplementary Fig. 2d. Theory and experimental response due to the drive tone in Supplementary Fig. 2c are in good agreement.

Characterization of RX qubit - coupling resonator coupling

We pointed out in the main text that the RX qubit-coupling resonator interaction strength g_{RX} decreases with decreasing RX qubit detuning Δ. g_{RX} can directly be extracted from the measurements in Supplementary Figs. 3b-e. There we set Δ to the working points presented in Figs. 3d-g, adjust ν_{C} with Φ_{C} and tune RX qubit and coupling resonator on resonance by sweeping ϵ (see Supplementary Fig. 3a). Note that the transmon is detuned for this measurement. We observe avoided crossings in the $|S_{11}|$ reflectance spectra with increasing separation from Supplementary Fig. 3b to Supplementary Fig. 3e. As the separation is $\simeq 2g_{\mathrm{RX}}$ this indicates an increase in g_{RX} with Δ in agreement with the theoretically expected behavior.

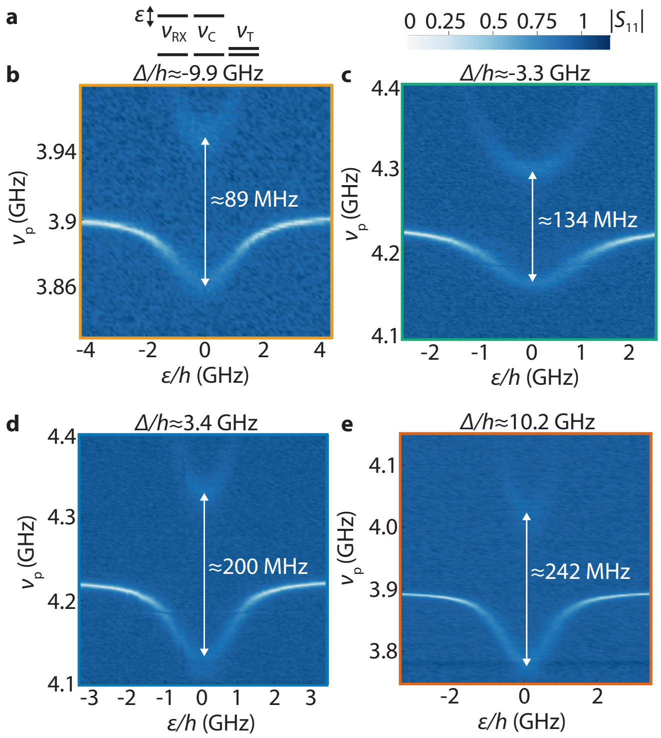

Supplementary Figure 3. Resonant RX-photon interaction at RX working points. **a** Schematic showing that for the measurements in **(b)-(e)**, RX qubit and coupling resonator are on resonance while the transmon is detuned energetically. **b-e** Spectrum of $|S_{11}|$ as a function of probe frequency ν_{p} and RX qubit asymmetry ϵ. The frame colors refer to Fig. 3: the RX qubit tunnel couplings and Δ-positions are the same as for the same colored working points in Fig. 3. Note that an offset in ϵ was added by hand such that the energy minimum is at $\epsilon \simeq 0$.

Resonant RX qubit-transmon-resonator interaction

As mentioned in the main text, the splitting of the dips in the reflection spectrum in Fig. 2d is enhanced for resonant RX qubit-transmon-resonator interaction at $\Delta \approx -7.8\,\mathrm{GHz}$ by $\approx 16\,\mathrm{MHz}$ compared to the situation where the transmon is detuned energetically from the hybridized resonator-transmon state ($\Delta/h \approx -11.4\,\mathrm{GHz}$). This enhancement is visible in transmission amplitudes $|S_{11}|$ measurements at both values of Δ which are extracted from Fig. 2d and shown in Supplementary Fig. 4.

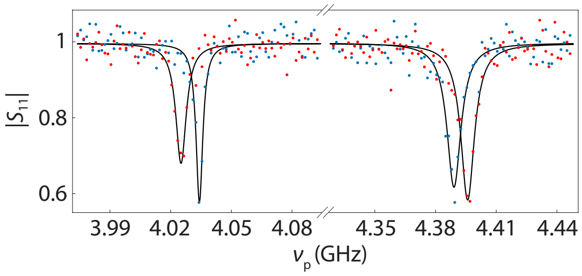

Supplementary Figure 4. Reflected amplitude $|S_{11}|$ as a function of probe frequency ν_{p}. The points indicate data extracted from Fig. 2d at $\Delta/h \approx -11.43\,\mathrm{GHz}$ (blue) and at $\Delta/h \approx -7.84\,\mathrm{GHz}$ (red). The solid lines are fits to a Lorentzian line shape.

Additional virtual interaction measurements

In this section we present data for virtual-photon-mediated RX qubit-transmon interaction in addition to the data presented in Fig. 3.

Thereby we choose the same RX qubit working points as in Figs. 3d-g. The measurement scheme in Supplementary Fig. 5a is almost identical to Fig. 3c: while RX qubit and transmon were tuned on resonance in Figs. 3d-g by sweeping the RX qubit energy with Δ, we instead tune to the resonance condition with Φ_{T} in Supplementary Fig. 5b. In Figs. 5c-d we repeat the measurements presented in Figs. 3d-g, however apply the drive tone via the resonators instead of driving the RX qubit via one of its gate lines.

The data in Fig. 3e and Supplementary Fig. 5b is very similar, as it is not relevant whether Δ or Φ_{T} is swept

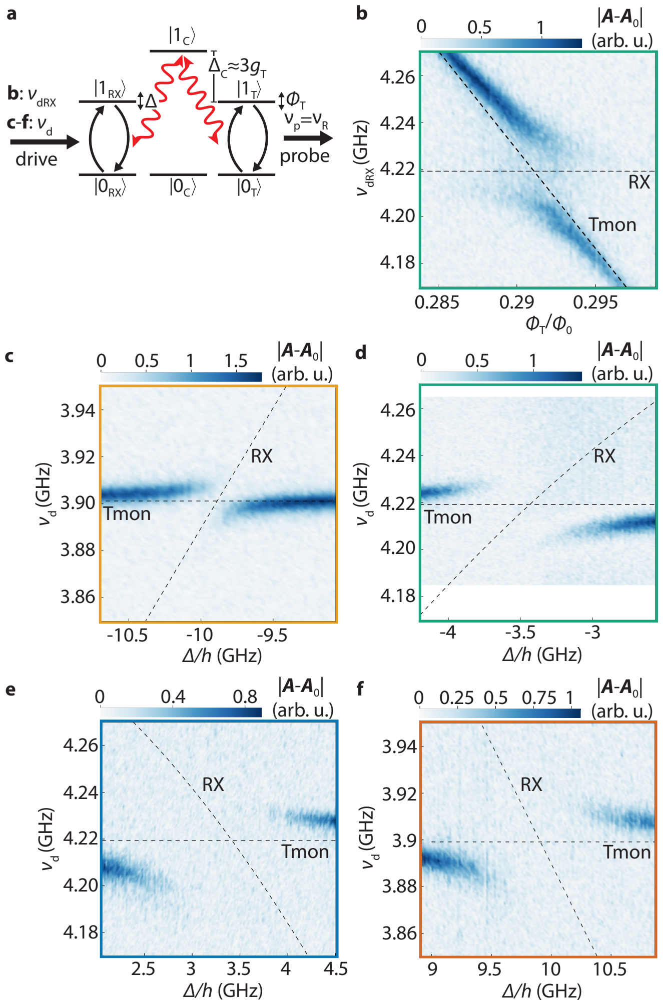

Supplementary Figure 5. Additional data on virtual RX-transmon interaction. The frame colors refer to the RX qubit working points in Fig. 3. **a** Measurement schematic as in Fig. 3c for the measurements in **(b)-(f)**. In **(b)** the drive tone is applied via the RX qubit, while it is applied via the resonators in **(c)-(f)**. The probe tone is on resonance with the read-out resonator. **b** Complex amplitude change $|\boldsymbol{A}-\boldsymbol{A}_0|$ at $\nu_\mathrm{p}=\nu_\mathrm{R}=5.63\,\mathrm{GHz}$ as a function drive frequency ν_dRX and normalized transmon flux Φ_T/Φ_0. The measurement parameters are identical to Fig. 3e except for sweeping the transmon (Tmon) energy with Φ_T instead of tuning the RX qubit energy with Δ. **c-f** $|\boldsymbol{A}-\boldsymbol{A}_0|$ at $\nu_\mathrm{p}=\nu_\mathrm{R}\simeq 5.6\,\mathrm{GHz}$ as a function of ν_d and Δ. The measurements are identical to Figs. 3d-g except for applying the drive tone via the resonator instead of via the RX qubit.

to tune both qubits on resonance. In Figs. 5c-f, where we drive the transmon, the visibility of the spectroscopic signal decreases when approaching the two-qubit resonance condition with Δ as the coherence of the hybridized transmon-RX qubit system is reduced compared to the bare transmon coherence. The reduced visibility is in contrast to Figs. 3d-g, where the coherence of the hybrid system is improved compared to the bare RX qubit coherence off resonance. By increasing the drive power in Supplementary Fig. 5, we expect the visibility on resonance to increase however at the cost adding power broadening to the system.

SUPPLEMENTARY NOTE 2: CIRCUIT QED THEORY

For simplicity, in the following section we used the notation $\hbar = 1$.

The dissipative dynamics of the system will be described by the master equation

$$\dot{\rho} = -\mathrm{i}\,[H,\rho] + \sum_k \mathcal{L}_k \rho \tag{1}$$

where H is the total Hamiltonian of the system and the $\mathcal{L}_k\rho$ describes different dissipative channels.

RX spin qubit

To derive the RX qubit Hamiltonian, we follow Refs. 3, 1 and 2 and consider the following three electron states within the $S_z = 1/2$ subspace:

$$\begin{aligned}
|0\rangle &= \frac{1}{\sqrt{6}}\left(|\uparrow,\uparrow,\downarrow\rangle + |\downarrow,\uparrow,\uparrow\rangle\right) - \sqrt{\frac{2}{3}}\,|\uparrow,\downarrow,\uparrow\rangle\,,\\
|1\rangle &= \frac{1}{\sqrt{2}}\left(|\uparrow,\uparrow,\downarrow\rangle - |\downarrow,\uparrow,\uparrow\rangle\right)\,,\\
|2\rangle &= \frac{1}{\sqrt{3}}\left(|\uparrow,\uparrow,\downarrow\rangle + |\downarrow,\uparrow,\uparrow\rangle\right) + \sqrt{\frac{1}{3}}\,|\uparrow,\downarrow,\uparrow\rangle\,,\\
|3\rangle &= \frac{1}{\sqrt{2}}\left(|\uparrow\downarrow\rangle_1 - |\downarrow\uparrow\rangle_1\right)|\cdot\rangle_2\,|\uparrow\rangle_3\,,\\
|4\rangle &= \frac{1}{\sqrt{2}}\,|\uparrow\rangle_1\,|\cdot\rangle_2\left(|\uparrow\downarrow\rangle_3 - |\downarrow\uparrow\rangle_3\right).
\end{aligned} \tag{2}$$

Here, the arrows denote the electron spin state in each dot, $|\uparrow\downarrow\rangle_k - |\downarrow\uparrow\rangle_k$ indicates the singlet state of two electrons in dot k and $|\cdot\rangle_k$ is the empty state of dot k. The states $|0\rangle$ and $|1\rangle$ with symmetric $(1,1,1)$ charge configurations as well as the states $|3\rangle$ and $|4\rangle$ with asymmetric $(2,0,1)$ and $(1,0,2)$ charge configurations have $S = S_z = 1/2$. They are tunnel coupled with amplitudes t_l and t_r. $|2\rangle$ is a leakage state with total spin $S = 3/2$ and $S_z = 1/2$, which does not interact with the $S = 1/2$ states via tunneling. In the basis from Eq. 2, the Hamiltonian can be written as:

$$H_0 = \begin{pmatrix}
0 & 0 & 0 & -\frac{\sqrt{3}}{2}t_\mathrm{r} & \frac{\sqrt{3}}{2}t_\mathrm{l}\\
0 & 0 & 0 & \frac{t_\mathrm{r}}{2} & \frac{t_\mathrm{l}}{2}\\
0 & 0 & 0 & 0 & 0\\
-\frac{\sqrt{3}}{2}t_\mathrm{r} & \frac{t_\mathrm{r}}{2} & 0 & -\varepsilon-\Delta & 0\\
\frac{\sqrt{3}}{2}t_\mathrm{l} & \frac{t_\mathrm{l}}{2} & 0 & 0 & \varepsilon-\Delta
\end{pmatrix}, \tag{3}$$

where $\varepsilon = (\varepsilon_3 - \varepsilon_1)/2$ and $\Delta = \varepsilon_2 - (\varepsilon_1 + \varepsilon_3)/2$. Thereby, ε_i is the energy of the state $(2,0,1)$ for $i = 1$, $(1,1,1)$ for

$i = 2$ and $(1, 0, 2)$ for $i = 3$. The leakage state is omitted in the following but is relevant again in Supplementary Note 3. By numerical diagonalization of Eq. 3 and omitting all but the two lowest lying eigenstates, we obtain the RX qubit Hamiltonian

$$H_{\mathrm{RX}} = -\frac{1}{2}\omega_{\mathrm{RX}}\sigma_z \tag{4}$$

where ω_{RX} is the RX qubit level splitting and $\sigma_z = |0_{\mathrm{RX}}\rangle\langle 0_{\mathrm{RX}}| - |1_{\mathrm{RX}}\rangle\langle 1_{\mathrm{RX}}|$ is a Pauli matrix in the basis of the RX qubit ground and excited states $|0_{\mathrm{RX}}\rangle$ and $|1_{\mathrm{RX}}\rangle$, respectively. Note that for $\Delta \ll 0$, $|0_{\mathrm{RX}}\rangle \simeq |0\rangle$ and $|1_{\mathrm{RX}}\rangle \simeq |1\rangle$.

The interaction with the coupling resonator then is of the form

$$H_{\text{RX-res}} = g_{\mathrm{RX}}\left(\sigma_- a^\dagger + \sigma_+ a\right) \tag{5}$$

where we performed a rotating wave approximation in the coupling, assuming $g_{\mathrm{RX}} \ll \omega_{RX}, \omega_C$, with the resonator frequency ω_C. Here, a is a bosonic annihilation operator of the resonator.

To take into account the dependence of the coupling strength g_{RX} on the parameters of the RX qubit, especially its changes with variations in Δ and ϵ, we use the expression derived in [2]

$$g_{\mathrm{RX}} = 2g_{\mathrm{charge,RX}}\left[c_3^{(1)}c_3^{(0)*} - \alpha\left(c_4^{(1)}c_4^{(0)*} + c_3^{(1)}c_3^{(0)*}\right)\right], \tag{6}$$

where the coefficients are defined from the eigenstates as

$$|0/1_{\mathrm{RX}}\rangle = \sum_{k=0}^{4} c_k^{(0/1)}|k\rangle, \tag{7}$$

and $g_{\mathrm{charge,RX}}$ is the charge coupling strength for the specific combination of tunnel couplings used in experiments.

Transmon

We model the transmon as a multilevel superconducting qubit with the Hamiltonian

$$H_{\mathrm{transmon}} = \sum_{k=0}^{N}\omega_k\sigma_{k,k} \tag{8}$$

where ω_k is the energy of the transmon eigenstate $|k\rangle$ and we introduced the operators $\sigma_{k,l} = |k\rangle\langle l|$. The energy levels ω_k are calculated from the full circuit model of the transmon [4], taking into account the experimental parameters charging energy E_{c}, maximum Josephson energy $E_{\mathrm{J,max}}$ and external flux through the SQUID loop Φ_{T}.

The transmon is coupled to both microwave resonators through the standard Jaynes-Cummings interaction for a multilevel system [5, 6]

$$H_{\text{trans-res}} = \sum_{j=\{R,T\}}\sum_{k=0}^{N} g_j^{(k)}\left(\sigma_{k,k+1}a_j^\dagger + \sigma_{k+1,k}a_j\right) \tag{9}$$

where $g_j^{(k)} \approx \sqrt{k+1}\,g_j^0$ and a_j is an annihilation operator for resonator j, and g_j^0 is the coupling strength between the two lowest levels of the transmon and either of the resonators (g_{T}^0 for coupling resonator and g_{R}^0 for read-out resonator). As the nature of the eigenstates changes slightly with changes in the external flux Φ_{T}, we always normalize the coupling strength in our calculations to the coupling at zero external flux (g_{T}^0 and g_{R}^0), using the expressions for the eigenstates we obtain from the full circuit model.

Typically in our calculations we use a cutoff for the number of transmon levels of $N = 3$, as we have checked that higher levels are not important for the dynamics considered here.

Coupled system

The full system is described by the transmon, RX qubit and the coupling and read-out resonators. Each of the resonators has the Hamiltonian

$$H_{\mathrm{res},j} = \omega_j a_j^\dagger a_j, \tag{10}$$

with its resonance frequency ω_j and where a_j is a bosonic annihilation operator, such that the total system Hamiltonian is

$$\begin{aligned} H_{\mathrm{total}} =& H_{\mathrm{RX}} + H_{\mathrm{transmon}} + \sum_j H_{\mathrm{res,j}} \\ &+ H_{\text{RX-res}} + H_{\text{trans-res}}. \end{aligned} \tag{11}$$

Depending on the part of the experiment considered, the simulations were performed only taking into account the relevant parts of the system. E.g. when fitting the interaction between transmon and read-out resonator, for simplicity the RX qubit and coupling resonator were not included in the model, as they were biased far detuned where they would not have any influence on the simulations.

Dissipative interactions

The RX qubit interactions with its environment consist of charge fluctuations in the leads as well as environmental magnetic field fluctuations.

We describe the dissipative processes through the master equation contributions

$$\mathcal{L}_{\mathrm{RX}}\rho = \gamma_{\downarrow,\mathrm{RX}}\mathcal{D}[\sigma_-]\rho + \frac{1}{2}\gamma_{\varphi,\mathrm{RX}}\mathcal{D}[\sigma_z]\rho \tag{12}$$

with the decay rate into its ground state $\gamma_{\downarrow,\mathrm{RX}}$ and its pure dephasing rate $\gamma_{\varphi,\mathrm{RX}}$. The linewidth observed in experiments is then given by the RX qubit's overall dephasing rate $\gamma_{2,\mathrm{RX}} = \frac{1}{2}\gamma_{\downarrow,\mathrm{RX}} + \gamma_{\varphi,\mathrm{RX}}$. For simplicity, we assume that the pure dephasing rate originates purely from the magnetic noise contribution that is discussed in detail in Supplementary Note 3. The remaining excess noise is caused by ohmic charge noise in the TQD gate electrodes. In this case we can write the RX decay rate as

$$\gamma_{\downarrow,\mathrm{RX}} = \beta\, \omega_{\mathrm{RX}} \left| \frac{g_{\mathrm{RX}}}{g_{\mathrm{charge,RX}}} \right|^2 \tag{13}$$

where β is a proportionality factor that encodes the noise spectral power and the absolute magnitude of its coupling to the RX qubit, ω_{RX} is the level splitting of the RX qubit and the factor $\left|\frac{g_{\mathrm{RX}}}{g_{\mathrm{charge,RX}}}\right|^2$ ensures the proper scaling with bias parameters for the charge noise coupling.

The transmon is subject to decoherence from its solid-state environment [5, 7, 8], leading to relaxation of its excited state into its ground state as well as pure dephasing. We model these processes through the incoherent dissipators

$$\mathcal{L}_{\mathrm{transmon}}\rho = \sum_k \gamma_{\downarrow,k}\mathcal{D}[\sigma_{k,k+1}]\rho + \gamma_\varphi \mathcal{D}[\sum_k \sigma_{k,k}]\rho\,. \tag{14}$$

where $\gamma_{\downarrow,k} \approx \left|\frac{g_k}{g_0}\right|^2 \gamma_{\downarrow,0}$. In practice, for the data available in this study, only the total linewidth of the transmon can be determined, and we cannot independently determine the decay rates $\gamma_{\downarrow,k}$ and dephasing rate γ_φ. Therefore we neglect the decay rates and only fit to the total linewidth $\gamma_{2,\mathrm{T}} = \gamma_\varphi$, which we assume to be roughly constant over the region of interest.

Each of the resonators is coupled to an environment, leading to incoherent photon loss at the rate κ_j, described in the master equation through a dissipative term

$$\mathcal{L}_{res}\rho = \sum_j \kappa_j \mathcal{D}[a_j]\rho. \tag{15}$$

In practice, the total resonator decay has an internal component κ_{int}, stemming from coupling to the intrinsic environment, and an external coupling rate, κ_{ext}, stemming from coupling to external modes, like the transmission lines used for driving the resonators. Here, the external coupling will be taken into account through the SLH cascading of an external driving field, described in the next section, so this part here only contains the intrinsic losses κ_{int}.

SLH scattering - spectroscopy

We use the SLH cascaded quantum systems approach to model scattering of photons off the two resonators [9–11]. Here, we cascade in a drive field for the probed resonator, which has the effect that it adds an effective drive Hamiltonian to the system

$$H_{\mathrm{drive}} = \frac{1}{2\mathrm{i}}\sqrt{\frac{\kappa_{\mathrm{ext}}}{2}} \sum_m \left(\alpha_m a^\dagger - \alpha_m^* a\right), \tag{16}$$

where we assumed a two-sided cavity which can be driven from both sides with either α_{R} or α_{L}. Here we have additionally transformed the system into the rotating frame at the drive frequency of the coherent field input α_m.

The cascading also adds another dissipative part to the master equation, which describes the decay of the resonator modes into the drive field (assumed to be a transmission line with infinite, constant spectrum)

$$\mathcal{L}_{\mathrm{SLH}}\rho = \mathcal{D}[\hat{L}_{\mathrm{R}}]\rho + \mathcal{D}[\hat{L}_{\mathrm{L}}]\rho \tag{17}$$

with the operators

$$\hat{L}_m = \sqrt{\frac{\kappa_{\mathrm{ext}}}{2}}\, a + \alpha_m \mathbb{1}\,. \tag{18}$$

We can now calculate the amplitudes and photon fluxes of the fields scattered off the resonator as

$$\beta_m = \mathrm{Tr}\{L_m\rho\} \quad , \quad n_m = \mathrm{Tr}\{L_m^\dagger L_m \rho\} \tag{19}$$

where ρ is the solution of the total master equation, including the drive and decay term from the cascading procedure. For simplicity we assume that scattering in experiments happens in the steady-state of the system, so that we need to calculate only the steady-state density matrix $\bar{\rho}$ for all cases.

Two-tone spectroscopy

Simulating two-tone spectroscopy is numerically challenging, since the Hamiltonian of the system will be explicitly time-dependent and there is no more steady-state. Instead of tackling this problem using time-dependent simulations or the more involved Floquet formalism for multiple driving fields, we note that for small amplitudes, in the linear response regime, the signal obtained from two-tone spectroscopy will be proportional to the single frequency response we calculate with the SLH formalism. We therefore still employ the SLH, single drive technique introduced above and for all fits introduce an additional scale factor to match experimental two-tone spectroscopy data.

Notes on fitting procedure

The fitting to obtain theory parameters from data generally followed a three step procedure. In a first step we fit the data to Lorentzians to extract the position of any peaks present in the measurements. We then use the

position of these peaks to extract the parameters of the relevant system Hamiltonians. Finally, where necessary when fitting to linecuts and transmission data directly, we then use the full SLH master equation simulation to extract linewidths. In any of these steps, when relevant, we adjust previously obtained parameters to better fit the data. This is necessary, as small drifts in parameters are unavoidable for solid state devices [7].

In the fits for the virtual interaction anticrossings in Figs. 3d-h, it turned out that there was significant power broadening present in the experiments. This manifested itself in a reduced size of the observed anticrossing to the expected value when applying the parameters obtained in previous fits, c.f. Fig. 2. As our fitting procedure, first adjusting Hamiltonian parameters using observed peak positions and then fitting the linewidth, cannot account for this kind of power broadening by itself, we adapted the fits to adjust the observed coupling strength between the RX qubit and the coupling resonator ($g_{\mathrm{charge,RX}}$) to account for the observed values. Additionally, when subsequently fitting the linewidths, we had to introduce significant additional broadening to the transmon linewidth $\gamma_{2,\mathrm{T}}$, for simplicity setting it equal to the RX linewidth $\gamma_{2,\mathrm{RX}}$ obtained in Fig. 3a. This combination lead to excellent quantitative agreement of the theory with the data.

Tables with the fit parameters of the theory curves in Figs. 1-3 can be found in Supplementary Note 4.

SUPPLEMENTARY NOTE 3: MAGNETIC-NOISE INDUCED DEPHASING

Assuming without any loss of generality that the RX qubit is defined in the $S_z = 1/2$ subspace, the decoherence effects due to magnetic noise can be divided into two different channels: leakage towards the $S_z = -1/2$ subspace, associated with in-plane magnetic field fluctuations, and decoherence within the $S_z = 1/2$ subspace of the qubit, associated with magnetic field fluctuations parallel to the quantization axis.

Decoherence within the $S_z = 1/2$ subspace

In this section we analyze the effects of leakage within the $S_z = 1/2$ subspace subspace, which, as will be shown, is the leading source of magnetically-induced decoherence. For the evaluation of the dephasing rate due to this mechanism we follow Refs. 12 and 13. We define the local differences in the magnetic field due to the hyperfine interaction in the left (1), middle (2) and right (3) quantum dot as $\delta B_\mathrm{l} = B_1^z - B_2^z$ and $\delta B_\mathrm{r} = B_2^z - B_3^z$. In these terms, the Hamiltonian due to magnetic field fluctuations is

$$H_{\delta B_z} = \frac{1}{2} g\mu_B \begin{pmatrix} \frac{2}{3}(\delta B_\mathrm{l} - \delta B_\mathrm{r}) & \frac{1}{\sqrt{3}}(\delta B_\mathrm{l} + \delta B_\mathrm{r}) & -\frac{\sqrt{2}}{3}(\delta B_\mathrm{l} - \delta B_\mathrm{r}) & 0 & 0 \\ \frac{1}{\sqrt{3}}(\delta B_\mathrm{l} + \delta B_\mathrm{r}) & 0 & \sqrt{\frac{2}{3}}(\delta B_\mathrm{l} + \delta B_\mathrm{r}) & 0 & 0 \\ -\frac{\sqrt{2}}{3}(\delta B_\mathrm{l} - \delta B_\mathrm{r}) & \sqrt{\frac{2}{3}}(\delta B_\mathrm{l} + \delta B_\mathrm{r}) & \frac{1}{3}(\delta B_\mathrm{l} - \delta B_\mathrm{r}) & 0 & 0 \\ 0 & 0 & 0 & -\delta B_\mathrm{r} & 0 \\ 0 & 0 & 0 & 0 & \delta B_\mathrm{l} \end{pmatrix}, \quad (20)$$

for the basis set defined in Eq. (2).

We assume the magnetic field fluctuations follow a Gaussian probability distribution such as

$$P(\delta B_\mathrm{l}, \delta B_\mathrm{r}) = \frac{1}{2\pi\sigma_B^2} e^{-(\delta B_\mathrm{l}^2 + \delta B_\mathrm{r}^2)/(2\sigma_B^2)}, \quad (21)$$

where σ_B is the standard deviation of the magnetic noise, which is assumed to be equal for all three dots. To calculate the decoherence rate due to the hyperfine interaction $\gamma_{2,\mathrm{RX}}^{(\mathrm{h})}$ we assume that the hyperfine perturbation of the qubit dynamics is static for a given evolution period, and we therefore solve the perturbed Schrödinger equation

$$-i\hbar\partial_t \rho = [\tilde{H}_0 + \tilde{H}_{\delta B_z}, \rho], \quad (22)$$

where the tilde above H_0 and $H_{\delta B_z}$ indicates that we have performed a transformation to the basis that diagonalizes the unperturbed Hamiltonian H_0, as defined in Eq. (3) at the relevant RX qubit working point. To evaluate the decay of the coherence, we assume the following initial state

$$|\psi_0\rangle = \frac{1}{\sqrt{2}}(|0\rangle + |1\rangle). \quad (23)$$

We now assume that the noise due to fluctuating hyperfine fields δB_l and δB_r is uncorrelated. Therefore it is possible to average the value of the coherence using the probability distribution given in Eq. (21). Hence, we obtain for the average value of the density matrix:

$$\langle\rho(t)\rangle = \int \frac{d(\delta B_\mathrm{l})d(\delta B_\mathrm{r})}{2\pi\sigma_B^2} \rho(t, \delta B_\mathrm{l}, \delta B_\mathrm{r}) e^{-(\delta B_\mathrm{l}^2 + \delta B_\mathrm{r}^2)/2\sigma_B^2} \quad (24)$$

The value of $\gamma_{2,\mathrm{RX}}^{(\mathrm{h})}$ is then determined by Gaussian decay of the coherence term in the density matrix:

$$|\langle\rho_{01}\rangle| = \frac{1}{2} e^{-(\gamma_{2,\mathrm{RX}}^{(\mathrm{h})} t)^2}. \quad (25)$$

Below, we first derive an analytical formula for the decoherence rate under the assumption that it mainly arises due to pure dephasing within the qubit subspace. For simplicity, we also assume that $t_\mathrm{l} = t_\mathrm{r} = t$. Later, we show how the numerical results based on Eq. (24) validate the assumptions made.
For a given working point we can approximate the qubit dynamics using the two-state Hamiltonian

$$H = -\frac{\hbar\omega_\mathrm{RX}}{2}\sigma_z + \big(h_\mathrm{l}(\Delta, t)\delta B_\mathrm{l} + h_\mathrm{r}(\Delta, t)\delta B_\mathrm{r}\big)\sigma_z, \quad (26)$$

where ω_RX is the RX qubit frequency at the working point, and $h_\mathrm{l,r}$ are the prefactors of δB_l and δB_r in the basis that diagonalizes H_0, after projecting onto the qubit subspace. These prefactors can be calculated analytically along the $\varepsilon = 0$ axis for a given Δ and t, giving $h_\mathrm{l} = -h_\mathrm{r}$ with

$$h_\mathrm{l} = \frac{g\mu_B}{24}\Big[-4 + \Delta\Big(\frac{3}{\sqrt{2t^2+\Delta^2}} + \frac{1}{\sqrt{6t^2+\Delta^2}}\Big)\Big]. \quad (27)$$

Inserting this two-level Hamiltonian in Eq. (22), and applying Eqs. (22)-(25) we get

$$\gamma_{2,\mathrm{RX}}^{(\mathrm{h})} = \frac{\sqrt{h_\mathrm{l}^2 + h_\mathrm{r}^2}\sigma_B}{\sqrt{2}\hbar}. \quad (28)$$

Applying Eq. (27) to Eq. (28) gives the following expression

$$\gamma_{2,\mathrm{RX}}^{(\mathrm{h})} = \frac{|g|\mu_B\sigma_B}{24\hbar}\Big[4 - \Delta\Big(\frac{3}{\sqrt{\Delta^2+2t^2}} + \frac{1}{\sqrt{\Delta^2+6t^2}}\Big)\Big]. \quad (29)$$

The resulting dependence on Δ is shown as the black curve in Supplementary Fig. 6a.

Alternatively, we numerically evaluate the integral in Eq. (24) for the full five-dimensional basis set, following the procedure described in Ref. 13, allowing us to also obtain results for asymmetric tunnel couplings. The resulting numerical values of $\gamma_{2,\mathrm{RX}}^{(\mathrm{h})}$ as a function of Δ are shown in Supplementary Fig. 6a for the two different asymmetric tunnel coupling configurations corresponding to the two sets of black data points in Fig. 3a.

The numerical values show that the dependence on the asymmetry of the tunnel couplings is very small in the relevant regime $\Delta/h \leq -7.5\,\mathrm{GHz}$, where $\gamma_{2,\mathrm{RX}}^{(\mathrm{h})}$ gives the main contribution to $\gamma_{2,\mathrm{RX}}$. In this hyperfine dominated regime, the maximal relative error in using Eq. (29) is $\approx 5\%$. Hence we use the analytic equation for the fit in Fig. 3a and extract $\sigma_B = 3.48\,\mathrm{mT}$ which is within the expected range for GaAs quantum dots [13].

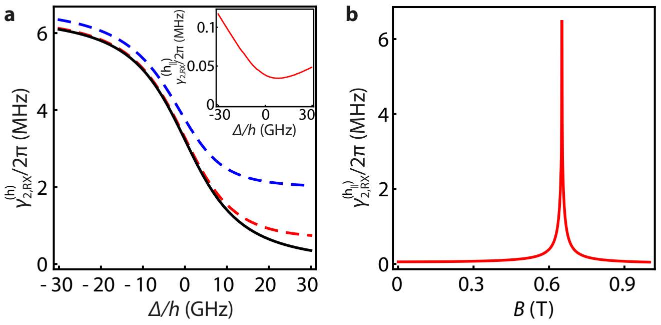

Supplementary Figure 6. Hyperfine induced decoherence. The graphs are obtained for $\sigma_B = 3.48\,\mathrm{mT}$ and $g = -0.4$. **a** Estimations of $\gamma_{2,\mathrm{RX}}^{(\mathrm{h})}$ as a function of Δ using Eq. (29) (solid) for $t/h = 9.03\,\mathrm{GHz}$, and the numeric calculations (dashed) for RX qubit tunnel coupling configurations 1 (blue) and 2 (red). Inset: Contribution $\gamma_{2,\mathrm{RX}}^{(\mathrm{h}_{||})}$ to decoherence from the in-plane magnetic field for RX qubit tunnel coupling configuration 2. **b** In-plane contribution as a function of the global field B for RX qubit tunnel coupling configuration 2. Note the peak around 0.651 T corresponds to the crossing between $|0^+\rangle$ and $|1^-\rangle$ states.

Leakage towards the $S_z = -1/2$ subspace

The in-plane magnetic field components only mix states with $S'_z = S_z \pm 1$. Leakage from the qubit subspace towards the opposite spin subspace is expected to be the dominating decoherence channel when the global magnetic field is suppressed [12]. However, since the cavity response does not depend on the spin subspace where the qubit is defined, the impact of this mechanism on the linewidth is non-trivial. For instance, the resonator mediates the interaction between qubit states, but there is no coupling between the pair of states with $S_z = +1/2$ and the pair of states with $S_z = -1/2$. This means that if there is a magnetic-noise-induced error that transforms the qubit state from $|0\rangle^+$ to $|0\rangle^-$, where the superscript $\pm$ indicates the spin subspace, the cavity response and the interactions mediated by the resonator would be unaffected. Alternatively, a bit flip from $|0\rangle^\pm$ to $|1\rangle^\mp$ is possible and has an impact on the cavity response linewidth. Fortunately, this mechanism is strongly suppressed at $B = 0$ due to the exchange interaction that splits the qubit states. To analyze this in-plane bit flip decoherence mechanism we consider the contribution of the in-plane components of the noisy magnetic field $(\delta B_x, \delta B_y)$. We then follow the same numerical procedure used to study fluctuations of the B_z field in Ref. 13. However here, at a given working point, only the bit-flip interaction terms are considered. In other words, the in-plane components only mix states with $S'_z = S_z \pm 1$. In this way, for the previously estimated value of $\sigma_B = 3.48\,\mathrm{mT}$, we estimate the value of the in-plane contribution to the hyperfine dephasing $\gamma_{2,\mathrm{RX}}^{(\mathrm{h}_{||})}$ in the inset of Supplementary Fig. 6a, finding that this mechanism is completely negligible.

Finally, in Supplementary Fig. 6b we estimate the effects of this decoherence mechanism as a function of the global magnetic field. The results show that the leakage only poses a problem when the $|0\rangle^\pm$ state is very similar in energy to the $|1\rangle^\mp$ state, over a narrow range of fields near $B \simeq 651\,\mathrm{mT}$.

Working point Δ/h [GHz]	RX qubit config.	ν_{RX} [GHz]	$\gamma_{2,\mathrm{RX}}/2\pi$ [MHz]	$g_{\mathrm{RX}}/2\pi$ [MHz]	ν_{C} [GHz]	$\kappa_{\mathrm{C}}/2\pi$ [MHz]	n	$\gamma_{\mathrm{P}}/2\pi$ [MHz]	$\gamma_{\mathrm{MID}}/2\pi$ [MHz]
$\simeq -9.9$	4	3901	8.7 ± 0.3	45.2	4519	4.1	0.04	0.02	0.09
$\simeq -9.9$	4	3903	8.3 ± 0.4	48.3	5164	5.0	0.03	0.01	0.11
$\simeq -3.3$	3	4225	11.9 ± 0.3	77.9	4845	5.1	0.01	0.08	0.10
$\simeq -3.3$	3	4228	7.8 ± 0.5	86.1	5895	9.2	0.04	0.02	0.36
$\simeq 3.4$	3	4221	22.9 ± 0.9	121.3	4845	7.0	0.04	0.24	0.55
$\simeq 3.4$	3	4222	14.8 ± 1.5	133.7	5894	6.9	0.02	0.04	0.25
$\simeq 10.2$	4	3871	28.3 ± 1.8	147.6	4541	7.0	0.04	0.30	0.55
$\simeq 10.2$	4	3895	18.9 ± 1.2	167.4	5902	7.9	0.02	0.05	0.29

Supplementary Table 1. Experimental parameters for decoherence measurement of RX qubit working points in Fig. 3a for small and large coupling resonator-qubit detuning. The coupling strength g_{RX} is obtained from theory using $g_{\mathrm{charge,RX}}$ as extracted from the Fig. 2a fit (see Supplementary Table 4). The average number n of photons in the coupling resonator is estimated from ac Stark shift measurements.

SUPPLEMENTARY NOTE 4: PARAMETER TABLES

Experimental parameters for RX qubit decoherence measurements

In Supplementary Table 1 we show relevant experimental parameters of coupling resonator and RX qubit that were used to determine $\gamma_{2,\mathrm{RX}}$ in Fig. 3a for the qubit working points. Thereby, $\gamma_{2,\mathrm{RX}}$ was determined both for small and large coupling resonator-qubit detuning. As discussed in the main text, we observe a significant increase in $\gamma_{2,\mathrm{RX}}$ at $\Delta > 0$ for small detuning. This increase cannot be explained by Purcell decay at rate γ_{P} [14] or measurement induce dephasing at rate γ_{MID} [15] as shown in Supplementary Table 1.

Theory fit parameters

In this section we quote fixed and free parameters of the theory fits that were used in the main text. Fixed fit parameters are marked with a star. They were either directly extracted from the experiment (e.g. resonance frequencies) or from previous fits (e.g. qubit parameters).

ω_{R} [GHz]	$g^0_{\mathrm{R}}/2\pi$ [MHz]	$E_{\mathrm{J,max}}$ [GHz]	E_{c} [GHz]
5.594 ± 0.002	141 ± 3	18.085 ± 0.282	0.221 ± 0.004

Supplementary Table 2. Fit parameters for theory curve in Fig. 1b.

RX config*	3
$g_{\mathrm{charge,RX}}/2\pi$ [MHz]*	186

Supplementary Table 3. Fit parameters for theory curve in Fig. 1e.

RX config.*	2
$\kappa_{\mathrm{C,int}}/2\pi$ [MHz]*	1.8
$\kappa_{\mathrm{C,ext}}/2\pi$ [MHz]*	2.8
ν_{C} [GHz]	4.229 ± 0.001
$g_{\mathrm{charge,RX}}/2\pi$ [MHz]	186 ± 2
$\gamma_{2,\mathrm{RX}}/2\pi$ [MHz]	15 ± 1

Supplementary Table 4. Fit parameters for theory curve in Fig. 2a and fit to black curve in Fig. 2c.

E_{c} [GHz]*	0.221
$E_{\mathrm{J,max}}$ [GHz]*	18.085
$\gamma_{2,\mathrm{T}}/2\pi$ [MHz]*	1.0
$\kappa_{\mathrm{C,int}}/2\pi$ [MHz]*	2.0
$\kappa_{\mathrm{C,ext}}/2\pi$ [MHz]*	2.6
ν_{C} [GHz]	4.175 ± 0.0003
$g^0_{\mathrm{T}}/2\pi$ [MHz]	205 ± 0.3

Supplementary Table 5. Fit parameters for theory curve in Fig. 2b and fit to green curve in Fig. 2c.

RX config.*	2
$\kappa_{\mathrm{C,int}}/2\pi$ [MHz]*	1.8
$\kappa_{\mathrm{C,ext}}/2\pi$ [MHz]*	2.8
$E_{\mathrm{J,max}}$ [GHz]*	18.085
E_{c} [GHz]*	0.221
$\gamma_{2,\mathrm{RX}}/2\pi$ [MHz]*	Fig. 3a fit
$\gamma_{2,\mathrm{T}}/2\pi$ [MHz]*	1.0
ν_{C} [GHz]	4.209 ± 0.0002
$g_{\mathrm{charge,RX}}/2\pi$ [MHz]	180 ± 2.0
$g^0_{\mathrm{T}}/2\pi$ [MHz]	203 ± 0.2
ν_{T}	ν_{C}
$2g_-/2\pi$ [MHz] @$\Delta/h = -9.77$ GHz	63 ± 1
$2g_+/2\pi$ [MHz] @$\Delta/h = -5.65$ GHz	84 ± 1

Supplementary Table 6. Fit parameters for theory curves in Figs. 2d-f.

RX config.*	2
σ_B [mT]	3.48 ± 0.06
β	31.45 ± 0.42

Supplementary Table 7. Fit parameters for theory curve in Fig. 3a.

$E_{\mathrm{J,max}}$ [GHz]*	18.085
E_c [GHz]*	0.221

Supplementary Table 8. Shared parameters for theory curves in Figs. 3d-h.

	f)	e)	remarks
RX config.*	3	3	
$\kappa_\mathrm{ext}/2\pi^*$ [MHz]	2.7	2.5	
$\kappa_\mathrm{int}/2\pi^*$ [MHz]	5.4	4.4	
ν_C [GHz]	4.764 ± 0.002	4.768 ± 0.001	
ν_T [GHz]	4.292 ± 0.002	4.292 ± 0.002	
$g_{\mathrm{charge,RX}}/2\pi$ [MHz]	113 ± 1	115 ± 1	
$g^0_\mathrm{T}/2\pi$ [MHz]	219 ± 2	219 ± 2	
$\gamma_{2,\mathrm{RX}}/2\pi$ [MHz]	15.2	9.7	from Fig. 3a fit
$\gamma_{2,\mathrm{T}}/2\pi$ [MHz]	15.2	9.7	power broadened
$2J/2\pi$ [MHz]	51.4	31.9	virtual splitting

Supplementary Table 9. Parameters for theory curves in Figs. 3e, f and associated linecuts in Fig. 3h.

	g)	d)	remarks
RX config.*	4	4	
$\kappa_\mathrm{ext}/2\pi^*$ [MHz]	3	3	
$\kappa_\mathrm{int}/2\pi^*$ [MHz]	4	4	
ν_C [GHz]	4.495 ± 0.004	4.481 ± 0.001	
ν_T [GHz]	3.958 ± 0.002	3.961 ± 0.002	
$g_{\mathrm{charge,RX}}/2\pi$ [MHz]	96 ± 2	101 ± 3	
$g^0_\mathrm{T}/2\pi$ [MHz]	213 ± 2	212 ± 2	
$\gamma_{2,\mathrm{RX}}/2\pi$ [MHz]	18.9	7.2	from Fig. 3a fit
$\gamma_{2,\mathrm{T}}/2\pi$ [MHz]	18.9	7.2	power broadened
$2J/2\pi$ [MHz]	49.2	14.9	virtual splitting

Supplementary Table 10. Parameters for theory curves in Figs. 3d, g and associated linecuts in Fig. 3h.

[1] Russ, M. & Burkard, G. Asymmetric resonant exchange qubit under the influence of electrical noise. *Phys. Rev. B* **91**, 235411 (2015).

[2] Landig, A. J. et al. Coherent spin–photon coupling using a resonant exchange qubit. *Nature* **560**, 179-184 (2018).

[3] Taylor, J. M., Srinivasa, V., Medford, J. Electrically Protected Resonant Exchange Qubits in Triple Quantum Dots. *Phys. Rev. Lett.* **111**, 050502 (2013).

[4] Koch, J. et al. Charge-insensitive qubit design derived from the Cooper pair box. *Phys. Rev. A* **76**, 042319 (2007).

[5] Blais, A. et al. Quantum-information processing with circuit quantum electrodynamics. *Phys. Rev. A* **75**, 032329 (2007).

[6] Slichter, D. H. et al. Quantum Zeno effect in the strong measurement regime of circuit quantum electrodynamics. *New J. Phys.* **18**, 053031 (2016).

[7] Müller, C., Lisenfeld, J., Shnirman, A., Poletto, S. Interacting two-level defects as sources of fluctuating high-frequency noise in superconducting circuits. *Phys. Rev. B* **92**, 035442 (2015).

[8] Schlör, S. et al. Correlating decoherence in transmon qubits: Low frequency noise by single fluctuators. Preprint at https://arxiv.org/abs/1901.05352 (2019).

[9] Combes, J., Kerckhoff, J., Sarovar, M. The SLH framework for modeling quantum input-output networks. *Adv. Phys. X* **2**, 784-888 (2017).

[10] Müller, C., Combes, J., Hamann, A. R., Fedorov, A., Stace, T. M. Nonreciprocal atomic scattering: A saturable, quantum Yagi-Uda antenna. *Phys. Rev. A* **96**, 053817 (2017).

[11] van Woerkom, D. J. et al. Microwave Photon-Mediated Interactions between Semiconductor Qubits. *Phys. Rev. X* **8**, 041018 (2018).

[12] Hung, J.-T., Fei, J., Friesen, M., Hu, X. Decoherence of an exchange qubit by hyperfine interaction. *Phys. Rev. B* **90**, 045308 (2014).

[13] Fei, J. et al. Characterizing gate operations near the sweet spot of an exchange-only qubit. *Phys. Rev. B* **91**, 205434 (2015).

[14] Sete, E. A., Gambetta, J. M., Korotkov, A. N. Purcell effect with microwave drive: Suppression of qubit relaxation rate. *Phys. Rev. B* **89**, 104516 (2014).

[15] Gambetta, J. et al. Qubit-photon interactions in a cavity: Measurement-induced dephasing and number splitting. *Phys. Rev. A* **74**, 042318 (2006).
